# Is Placental Mitochondrial Function a Regulator that Matches Fetal and Placental Growth to Maternal Nutrient Intake in the Mouse?

**DOI:** 10.1371/journal.pone.0130631

**Published:** 2015-07-01

**Authors:** Marcos R. Chiaratti, Sajida Malik, Alan Diot, Elizabeth Rapa, Lorna Macleod, Karl Morten, Manu Vatish, Richard Boyd, Joanna Poulton

**Affiliations:** 1 Nuffield Department of Obstetrics and Gynaecology, The Women’s Centre, University of Oxford, Oxford, OX3 9DU, United Kingdom; 2 Departamento de Genética e Evolução, Universidade Federal de São Carlos Rod. Washington Luís, km 235, Jardim Guanabara, São Carlos, SP, CEP 13.565–905, Brazil; 3 Department of Physiology Anatomy and Genetics, University of Oxford, Oxford, OX1 3QX, United Kingdom; University of Texas Health Science Center at San Antonio, UNITED STATES

## Abstract

**Background:**

Effective fetal growth requires adequate maternal nutrition coupled to active transport of nutrients across the placenta, which, in turn requires ATP. Epidemiological and experimental evidence has shown that impaired maternal nutrition in utero results in an adverse postnatal phenotype for the offspring. Placental mitochondrial function might link maternal food intake to fetal growth since impaired placental ATP production, in response to poor maternal nutrition, could be a pathway linking maternal food intake to reduced fetal growth.

**Method:**

We assessed the effects of maternal diet on placental water content, ATP levels and mitochondrial DNA (mtDNA) content in mice at embryonic (E) day 18 (E18). Females maintained on either low- (LPD) or normal- (NPD) protein diets were mated with NPD males.

**Results:**

To investigate the possibility of an underlying mitochondrial stress response, we studied cultured human trophoblast cells (BeWos). High throughput imaging showed that amino acid starvation induces changes in mitochondrial morphology that suggest stress-induced mitochondrial hyperfusion. This is a defensive response, believed to increase mitochondrial efficiency, that could underlie the increase in ATP observed in placenta.

**Conclusions:**

These findings reinforce the pathophysiological links between maternal diet and *conceptus* mitochondria, potentially contributing to metabolic programming. The quiet embryo hypothesis proposes that pre-implantation embryo survival is best served by a relatively low level of metabolism. This may extend to post-implantation trophoblast responses to nutrition.

## Introduction

The Dutch famine is perhaps the best known example [1] of a study showing that poor intrauterine nutrition may lead to adverse phenotypes in postnatal life, now well supported in both humans [2] and animals [3,4]. These adverse outcomes, including features of the metabolic syndrome, such as type 2 diabetes, are often linked to poor or disproportionate fetal growth: fetal growth restriction [5]. The molecular mechanisms mediating these effects are not clear [6], but evidence points to a critical role for mitochondria in both insulin secretion and in placental function.

Pancreatic β-cells require mitochondrial ATP production for glucose-stimulated insulin secretion [7], and are sensitive to defects in mitochondrial function. Thus, many mutations in mitochondrial tRNA genes are associated with diabetes [8], and Kearns-Sayre syndrome, which is due to mtDNA rearrangements, is associated with the development of small pancreatic islets [9] and with diabetes [10].

During pregnancy, active cellular transport across the placenta requires ATP, much of which is likely to be derived aerobically. While ATP is directly involved in some of these transport processes, most are driven by secondary active transport and glucose itself is not actively transported across the placenta. Hence reduced fetal growth might be linked through a final common pathway to impaired mitochondrial function [11] and placental ATP production, placental insufficiency [12], and hypoxia [12]. Thus, placental ATP production might be directly related to nutrient supply to the placenta, and if this was rate limiting for processes such as active transport thereby regulate fetal growth. Down-regulating mitochondrial function in response to poor nutrition could thus be an epigenetic mechanism (in its broadest sense) for long-term adaptation of gene function to prevailing environment.

Mitochondria could thus play a central role in metabolic programming. For example, we identified a mtDNA sequence variant that predisposes to both thinness in babies [13] and young adults [14], but to type 2 diabetes in multiple older populations [15] [16] [17]. Circumstantial evidence suggests that this variant may play a role in the “predictive adaptive response” [18] whereby mismatch between pre- and post-natal environment is an important determinant of health. This is because the mtDNA variant appears to exacerbate the effect of poor intra-uterine nutrition (manifest as catch-up growth) on both neonatal ponderal index and adult glucose tolerance, potentially by subtly impairing mitochondrial function. These findings led us to investigate the effect of low protein diet on placental mitochondrial function in mouse.

## Material and Methods

### Experimental design

In this study we assessed the effects of maternal diet on litter size (n = 9–12 for each condition) and, on fetal and placental wet and dry weights (n = 31–38 for each condition) in mice at embryonic (E) day 18 (E18). In addition, to investigate whether placental mitochondria are involved in poor fetal and placental growth, we measured mtDNA (n = 25–28 for each condition), ATP (n = 34–47 for each condition) and gene expression levels (n = 5 for each condition) in E18 mouse placentas.

### Animals and sampling

All animals were housed and managed in accordance with the United Kingdom’s Home Office protocols and with the approval of the University of Oxford Ethical Review Committee. The animals were eight week-old female C57BL/6 mice which were randomly allocated to one of two different regimens: an LPD (8% total protein, Hope Farms 4400.01, Woerden, Netherlands) or a normal-protein diet (NPD; 14% total protein, Hope Farms 4400.00). These were given *ad libitum* access to the diets and water from two weeks prior to mating to the end of pregnancy.

For matings, females from both groups were paired overnight with control C57BL/6 males (subjected to NPD), plugged, and the day after mating was designated E1. At E18, pregnant females were euthanized to recover pups and their placentas. After careful separation of both, pups and placentas (after removing membranes) were individually weighted, placed in different cryotubes and snap-frozen in liquid nitrogen. Samples were stored at -80°C until analysis. Pups and placenta samples were dried at 80°C for 24 h (by which time weight was stable) to assess dry weight. Alternatively, stored placentas were used to assess mtDNA and ATP content. Placental efficiency was defined as the fetal wet weight/placental wet weight on day E18.

### Measurement of adenosine triphosphate in placentas

The ATP Bioluminescence Assay Kit CLS II (Roche, Mannheim, Germany) was employed to measure ATP in mouse placentas, stored placenta samples being homogenized in 1.5 ml of boiling TE buffer (100 mM Tris, 4 mM EDTA, pH 7.75), as in the manufacturer’s recommendations. Then, 0.5 ml homogenate was incubated for 2 min at 100°C, immediately centrifuged at 1,000 X g for 1 min at 4°C. The supernatant was transferred to a new tube and kept on ice. The remaining tissue homogenate was used for protein and mtDNA quantification.

For protein quantification, 100 μl of the tissue homogenate was diluted in 200 μl of water and sonicated for 30 s on ice before centrifugation at 15,000 X g for 15 min at 4°C. The supernatant had total protein quantified (triplicate) by the Lowry method (Bio-Rad Laboratories, Hemel Hempstead, UK). When ATP and mtDNA content were assessed in different pieces of a single placenta, the placenta was divided into five pieces which were homogenized individually in 400 μl of boiling TE buffer. The homogenate (125 μl) was used for ATP measurement as described above. For protein quantification within a single placental piece, 25 μl of homogenate were diluted in 100 μl of water to be processed as described above.

To measure ATP in mouse placentas using the ATP Bioluminescence Assay Kit CLS II, a ten-fold serial-diluted standard curve of ATP (10 μM-1 nM) was freshly prepared (using ATP provided by the kit) and run in parallel with samples. All samples and standard curves were analysed in triplicate in a luminometer (Fluostar Optima, BMG Labtech, Offenburg, Germany). Samples were run in duplicate when ATP was measured within a single placental piece. Sample ATP content was estimated using the standard curve generated and corrected for the amount of protein from the same sample. At least 35 samples taken from a minimum of six different litters were considered for each experimental group. When ATP was assessed in five different pieces of a single placenta, at least five placentas were assessed from each experimental group.

### Relative quantification of placental mtDNA

Mouse samples had DNA extracted from 400 μl (or 175 μl when considering a single placental piece) of homogenized tissue (see above) using the DNeasy Blood & Tissue Kit (Qiagen, Hilden, Germany). Quantification of mtDNA (*mt-Nd5*) levels relative to nuclear DNA (*Mpg*) was performed using a quantitative real-time PCR (qPCR) method as previously described [19]. To ensure consistency, both mtDNA and nuclear DNA assays were always run in the same PCR plate. A total of 0.5 ng and 25 ng of sample DNA were used per reaction for the mtDNA and the nuclear DNA assays, respectively. For each run, a standard curve was generated using seven two-fold serial-dilutions of a pool of sample DNA. Based on the standard curve values, it was possible to estimate the starting amount of mtDNA relative to nuclear DNA. Each experimental group contained at least 25 samples taken from a minimum of six different litters. When mtDNA was assessed in five different pieces of a single placenta, at least five placentas were analysed from each experimental group.

### Relative quantification of imprinted gene expression

We investigated the expression levels of imprinted genes, involved in growth and development in the placenta and implicated in parent-of-origin effects by the “Conflict hypothesis” (suggesting that paternally inherited alleles function to produce large, well developed placenta and hence offspring, whereas maternally expressed genes conserve maternal resources for the future [20]. We hypothesized that maternally expressed genes would be more likely to respond to adverse nutrition that paternally expressed genes. The genes studied were i) *Ata3*, a maternally imprinted gene encoding a component of the amino acid transporter system expressed heavily in placenta; ii) *Ata2*, a non-imprinted subunit of the same transporter; iii) *Igf2r*, a paternally imprinted gene regulating the growth promoting effects of the maternally imprinted *Igf2*, which was also investigated; iv) *Peg3*, maternally imprinted, is involved in p53-mediated apoptosis downstream of p53 action; v) *Slc6a6*, an sodium- and chloride-dependent taurine transporter; and vi) *Zac1*, a zinc finger protein involved in apoptosis and cell cycle regulation.

RNA was extracted from placentae in RNABee (AMS Biotechnology) and reverse transcribed (Superscript III, Invitrogen). cDNA was diluted and q-PCR run on a GeneAmp 5700 machine (Applied Biosystems) using primers and probes for *Igf2*, *Igf2R*, *Peg3*, *Slc38a1*, *Slc38a2*, *Slc38a4*, *Slc6a6*, and *Zac1*, and the housekeeping genes *beta-actin* and *GAPDH* [21] (Applied Biosystems). Q-RT-PCR was performed on all 10 genes and standard curves were produced as described above for each. Changes in expression was measured by determining the ratio of selected gene expression:housekeeping gene expression.

Placental RNA was extracted from tissue using phenol based methods and cDNA synthesised and Real Time PCR performed to quantify expression of *Actb*, *Gapdh*, *Igf2*, *Igf2r*, *Peg3*, *Slc38a*, *1Slc38a2* and *Slc38a4*, *Slc6a6* and *Zac1* using Applied Biosystems’ inventoried assays. Gene expression was assessed relative to *Actb* and *Gapdh* and log transformed for statistical analysis.

### Cell biology methods

BeWos cells were cultured in a 96-well plate and treated for 6 hours in the indicated conditions before fixation with 4% paraformaldehyde (PFA). After DAPI and immuno-staining using a monoclonal antibody anti-Tom20 (Santa Cruz Biotechnology, mouse) and a polyclonal antibody anti-LC3 (Medical and Biological Laboratories, rabbit) combined with Alexa Fluor 488 Goat anti-rabbit and Alexa Fluor 568 Goat anti-mouse (life technologies) secondary antibodies the plate is imaged using the IN Cell 1000 analyser (GE healthcare life sciences, 500 cells per well). Raw images were binarised and mitochondrial morphological characteristics were quantified, notably the degree of branching or mitochondrial form factor (FF) and the mean mitochondria length (in μm). FF is defined as (Pm^2^)/(4πAm), where Pm is the length of mitochondrial outline and Am is the area of mitochondrion (Mortiboys et al., 2008), and with a value comprised between 1 (fragmented network in individual dots) and 0 (infinitely connected network). Mitochondria with a FF <0.8 are elongated and we calculated the proportion of these from the mitochondria identified.

### Statistical analysis

All experiments were repeated three times in their entirety except that in Run 1, placental dry weights were measured and hence not available for other measurements. Statistical analysis was performed using the IBM SPSS statistics package (version 22). For our initial analysis we used litter mean as the target variable. Experimental groups were compared by Student’s t-test. We followed up each litter-summarized analysis with a mixed-effects model analysis of individual data points with litter identity as a random effect.

For gene expression studies, we used log transformed response variables with litter identity as a random effect. Differences with p values less than 0.05 were considered significant. Values are reported as estimated difference between diets (on log scale) +/- standard error.

## Results

**Fig 1:**
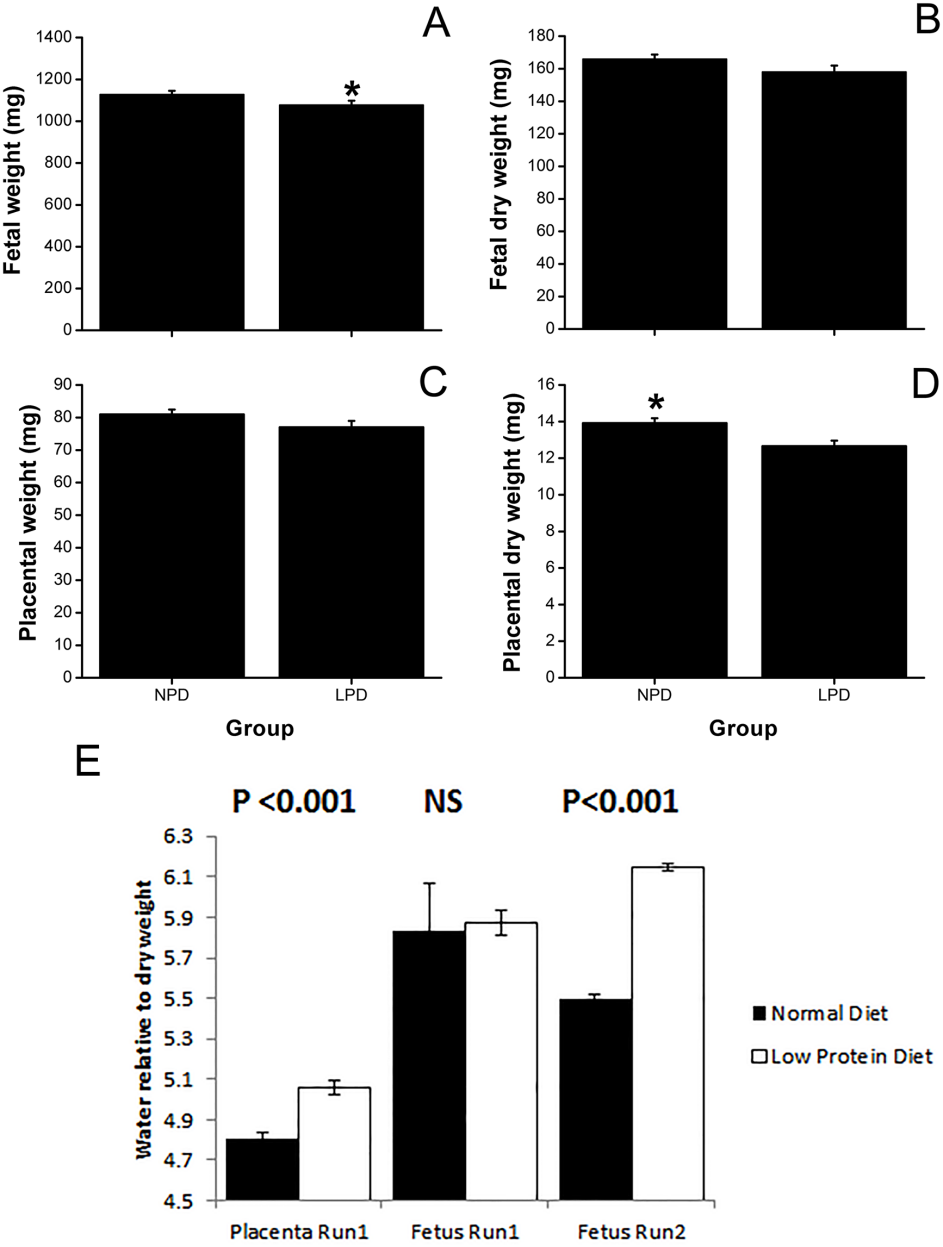
Low-protein diet (LPD) impairs fetal and placental growth compared with normal-protein diet (NPD). Fetal (A) and placental (C) fresh weights were recorded at day 18 of pregnancy from mothers subjected to NPD or LPD. These were re-weighed after drying (B and D respectively). These data are plotted as individual fetal (A, B) and placental (C, D) weights. Summed fetal and placental weights were not consistently different. Values are reported as means ± SEM. Part (E) shows the ratio of water to dry weight of individual fetuses or placentae then averaged by litter. The median litter value is plotted. This was substantially increased in Placenta (p<0.001). It was also increased in fetuses from run 2, (p<0.001) but not run 1. Error bars are SE *Difference between groups (P < 0.05). Parts A to D show Run 1, part E shows Runs 1 and 2.

The data presented is from three runs of the same experiment run on different occasions. Fig 1 refers to Run 1, where placental dry weights were measured and hence not available for other measurements. The final figure refers to Run 3 where placenta was used for cDNA analysis. All other figures refer to Run 2.

### LPD increases placental water content

Since cellular water content reflects the efficiency of ATP-dependent active transport [22,23], we assessed the effects of LPD on placental water content. Reduced oncotic pressure resulting from protein deficiency might also be associated with excess fluid.

Individual fetal fresh weight (NPD = 1127 ± 21 mg vs. LPD = 1078 ± 18 mg; P = 0.03), but not individual placental fresh weight (NPD = 81 ± 1.48 mg vs. LPD = 77 ± 1.97 mg; P = 0.16), was decreased by LPD (Fig 1). Yet, when individual placental dry weights were analysed, they were significantly reduced (NPD = 14 ± 0.27 mg vs. LPD = 13 ± 0.30; P = 0.003) but individual dry fetal weights were not. Litter size, summed litter fresh fetal and summed litter fresh placental weight were not consistently affected by LPD.

**Fig 2:**
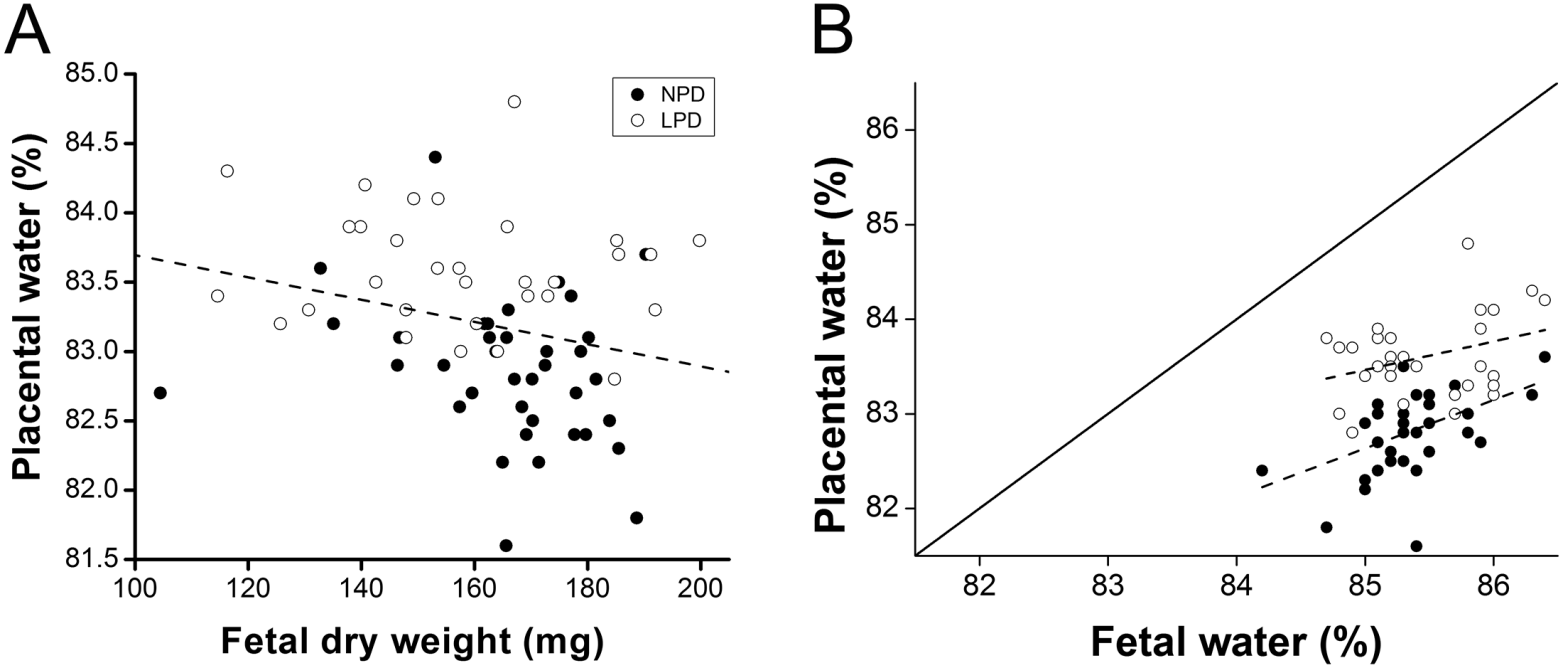
A) Fetal dry weight is inversely proportional to placental water and B) low fetal dry weight is associated with low placental efficiency (fetal/placental fresh weight) in mice. Weights were measured in day-18 (pregnancy) mouse placentas from mothers subjected to normal- (NPD) or low- (LPD) protein diets. Each dot is representative of a single placenta. The line represents the correlation between the two variables for (A) (r = -0.26, P = 0.03), (B) (NPD: r = 0.46, P = 0.006; LPD: r = 0.34, P = 0.07).

Metabolic energy expenditure plays a critical role in regulating cell volume, we used placental water content as an indicator of successful ion pumping, which of course underpins many aspects of trophoblast cell function. Placental water content correlated negatively with fetal dry weight (r = -0.26, P = 0.03; Fig 2A). It was significantly increased by LPD compared with NPD (83.6 ± 0.077% vs. 82.9 ± 0.087%; P = 0.0001). When expressed as the ratio of water content to dry weight (Fig 1E) this was substantially increased in placenta (p<0.001). It was also increased in fetuses from run 2 (p<0.001) but not run 1. Placental water content also correlated positively with fetal water content for the NPD (r = 0.46, P = 0.006), with a similar trend for the LPD litters (r = 0.34, P = 0.07; Fig 2B). We also found a positive correlation (r = 0.53, P = 0.0001) between fetal dry weight and placental efficiency (defined as fetal/placental fresh weight, not shown). The disadvantage to growth conferred by LPD persisted postnatally (S1 Fig). Having shown that LPD placentae had a higher water content, reflecting reduced efficiency of ATP-dependent active transport [22,23], we wished to test whether LPD placentas had a lower ATP and mtDNA content or altered expression of placental transporters compared to NPD.

### Placental ATP content was increased by LPD

**Fig 3:**
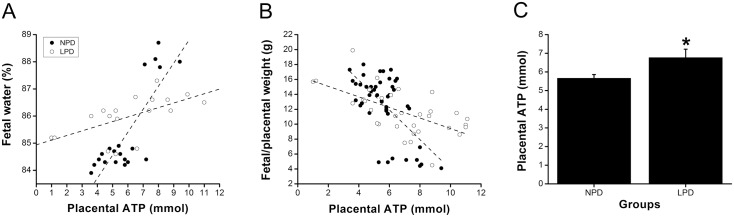
Placental ATP increases with fetal water content in mice and is inversely correlated to placenta efficiency (fetal/placental fresh weight). The content of ATP was measured in day18 (pregnancy) mouse placentas from mothers subjected to normal (NPD) or low (LPD) protein diets. Each dot is representative of a single placenta. In (A), the line represents the correlation between the two variables for NPD (r = 0.81, P = 0.0001) and LPD (r = 0.60, P = 0.025). In (B), the two variables were inversely correlated (r = -0.56, P = 0.0001). In (C), values are reported as means ± SEM and difference (P = 0.03) between groups is indicated by (*). The number of samples considered in (C) was 45 for the NPD and 34 for the LPD.

**Fig 4:**
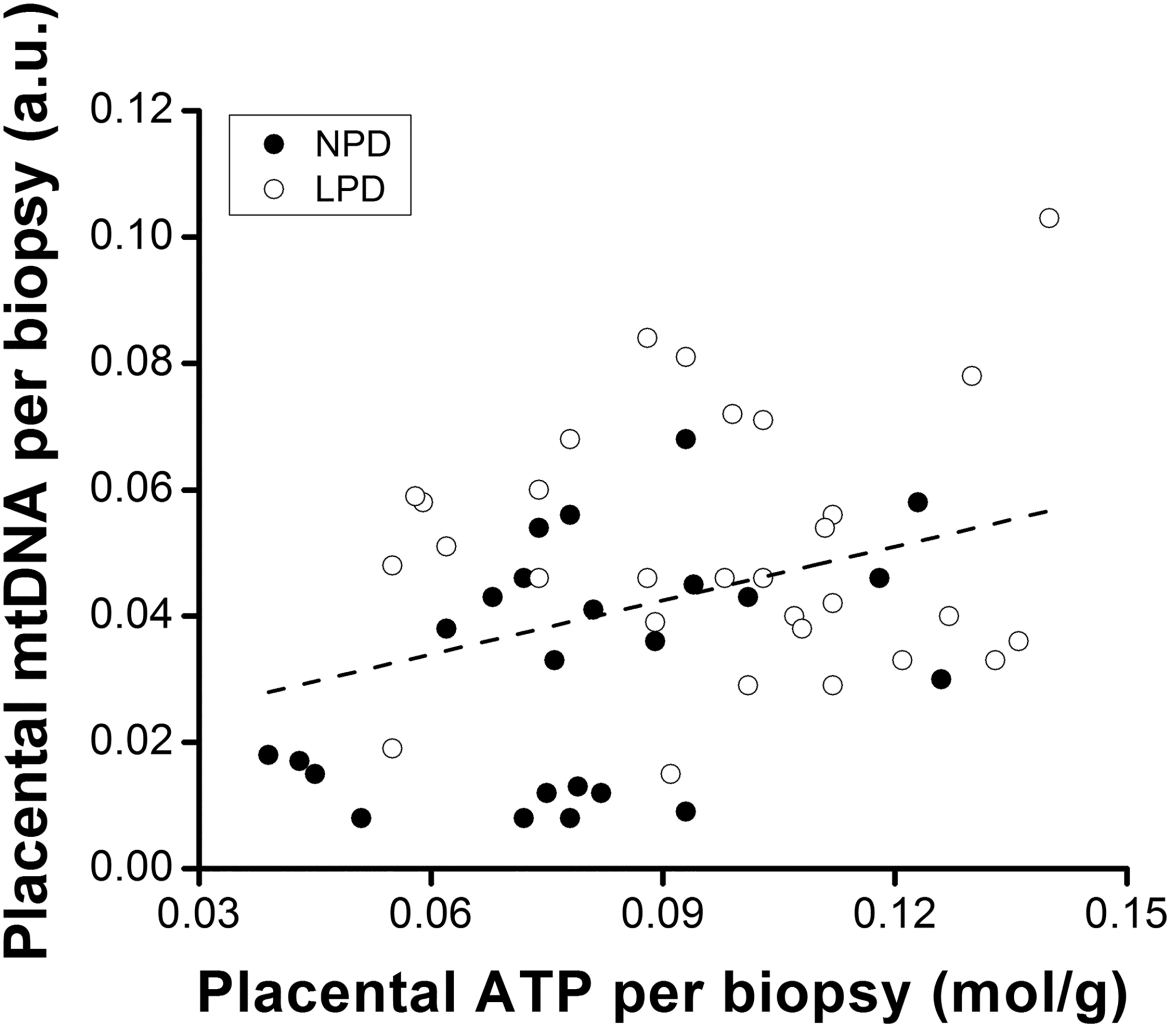
Placental mitochondrial DNA (mtDNA) was directly correlated with ATP levels when placenta is divided into small biopsies. The content of mtDNA per nuclear DNA and ATP concentration was measured in day-18 (pregnancy) mouse placentas from mothers subjected to normal- (NPD) or low- (LPD) protein diets. Each dot is representative of a single placental biopsy. The line represents the correlation between the two variables (r^2^ = 0.34, P = 0.01). There was no significant correlation when the biopsy values for each placenta were averaged. a.u. indicates arbitrary units.

**Fig 5:**
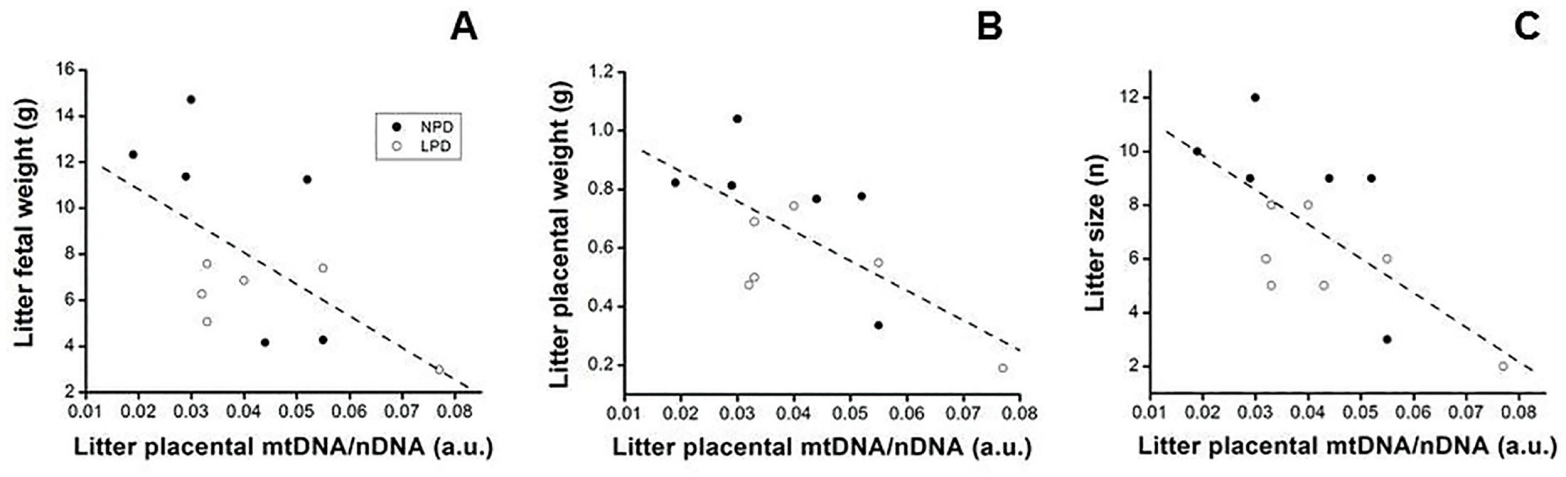
Placental mouse mitochondrial DNA (mtDNA) is highest in the least productive litters and in the smallest litters. The mean content of mtDNA per nuclear DNA was measured in day-18 (pregnancy) mouse placentas from mothers subjected to normal- (NPD) or low- (LPD) protein diets. Each dot is representative of a single litter. The line represents the correlation between the two variables for (A) (r = -0.58, P = 0.04), (B) (r = -0.68, P = 0.02) and (C) (r = -0.68, P = 0.01). a.u. indicates arbitrary units. Data is taken from Run 2, but Run 1 is comparable.

To investigate whether ATP and mtDNA content are linked as we predicted, we measured ATP and mtDNA (relative to nuclear DNA) levels in mouse placentas. Placental ATP content was not reduced in fetuses with a high water content (Fig 3A), but was inversely proportional (r = -0.56, P = 0.0001) to placental efficiency (Fig 3B). Furthermore, the placental ATP level was increased (P = 0.03) by the LPD (6.8 ± 0.453 mmol) compared with the NPD (5.7 ± 0.198 mmol; Fig 3C). Yet, placental mtDNA content (measured as the ratio of mtDNA to nuclear DNA) was not significantly increased (P = 0.82) in LPD (1.07 ± 0.089) compared to NPD (1.0 ± 0.087) placenta. Neither did total placental ATP and total mtDNA content correlate (not shown). Because we had previously found that mtDNA content varied by 3–4 fold within regions of a single placenta [24], we re-visited these data by dividing each placenta into five portions (each weighing 10–20 mg). In this case (Fig 4), a significant positive relationship between placental mtDNA/nuclear DNA and ATP became apparent (r = 0.34, P = 0.01) whereas mtDNA varied by 1.5–4 fold within several pieces of a single placenta. Furthermore, in both runs litter placental mtDNA content was inversely correlated to summed litter fresh weight (r = -0.59, P = 0.04; Fig 5A), summed litter placental fresh weight (r = -0.68, P = 0.02; Fig 5B) and litter size (r = -0.68, P = 0.01; Fig 5C).

### Expression of placental transporters was modulated by LPD

**Fig 6:**
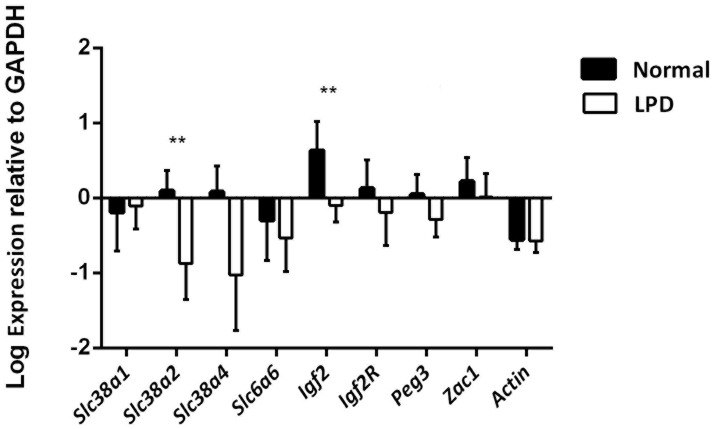
Expression of placental transporters *Slc38a1*, *Slc38a2*, *Slc38a4* and *Slc6a6* and of *Igf2*, *Igf2R*, *Peg3 and Zac1* in placenta at day 18 was assessed by reverse transcription of mRNA followed by Q-PCR, log transformed and corrected for expression of *GAPDH* and *Act* and expressed relative to controls. Plot shows means of corrected log transforms, error bars are standard errors using individual mice as the unit of analysis. The expression of *Slc38a2* and *Igf2* (p<0.009 and p<0.006 respectively) were significantly reduced by LPD. Key ** p<0.01.

To determine whether the increased water content in LPD fetuses reflected altered nutrient transport, we investigated the expression of a number or transporter using qPCR of cDNA. When individual litters were included as a random variable, *Slc38a2* and *Igf2* expression (relative to house-keeping gene *Gapdh* and log transformed) was significantly reduced in LPD placenta (p = 0.009 and p = 0.006 respectively, Fig 6). However, if considered as independent of litter, expression of placental transporters *Slc38a2* and *Slc38a4* and of factors *Igf2*, and *Peg3* was significantly reduced in LPD placenta (p<0.001, p = 0.001, 0.001 and 0.02, using individual mice as the unit of analysis. The latter reduction was not affected by the mtDNA copy number. The expression of *Slc38a1*, *Slc6a6*, *Igf2R* and *Zac1* was not significantly different from control.

### The response of placental mitochondria to stress

Because mitochondria are able to increase cellular ATP by the stress response, known as Stress Induced Mitochondrial Hyperfusion (SIMH) in response to nutrients [25], we studied the effect of various types of cellular starvation on an immortalised human trophoblast line (BeWo). We used high throughput imaging (IN Cell 1000) to determine the mitochondrial morphology. Mitochondria were significantly more elongated in BeWo cells starved of amino acids for three days compared to baseline (Fig 3B shows two representative runs). Similarly, this significantly decreased the proportion of rounded and of short mitochondria (p = 0.002 and p<0.001, T tests). As expected [25], mitochondrial fragmentation was apparent in several other types of nutrient deprivation (not shown).

## Discussion

We have shown that LPD (previously linked to insulin resistance in later life) increased placental water content. This effect was associated with reduced placental efficiency and expression of placental transporters, but with increased placental ATP content. We also showed that mitochondria, in human trophoblast cells that had been starved of amino acids, showed evidence of a stress response for raising cellular ATP.

These data show that combining simple measurements of placental or fetal dry and wet weights correlates with maternal diet and may reflect fetal nutrition that reflects post-natal growth and correlates with mtDNA content. They show that the detrimental effect of maternal LPD on placental efficiency is not due to a deficiency of either placental mtDNA or ATP. For the first time, using LPD and NPD we have shown that placental mtDNA is highest in the least successful litters, perhaps because mtDNA copy number increases in oxidative stress [26].

Fetal and placental water contents appear to be a useful measure of placental efficiency, being inversely proportional to dry weights. The placenta contains significantly less water content than does the fetus. Importantly, placental water content was highest for the fetuses with the lowest dry weights, suggesting that high water content is a sign of an unsuccessful potentially oedematous placenta. It would be interesting to confirm this by measuring sodium pump activity. Consistent with previous studies [27], expression of placental transporters *Slc38a2* and *Slc38a4* was reduced, potentially secondarily to reduced *Igf2* expression [28].

In contrast to our prediction that LPD might impair ATP synthesis, placental ATP content is increased by LPD. These data could be strengthened by measuring ADP and AMP concurrently. While ATP is very labile and *in vivo* values may well be higher, our data suggest that placental ATP production is not the final common pathway linking placental insufficiency, hypoxia and impaired placental mitochondrial function to reduced fetal growth. Other mechanisms such as epigenetic and environmental regulation of the placental phenotype by means of imprinted genes are clearly important [29]. It is well known that pre-implantation embryo survival is best served by a relatively low level of metabolism, the so-called “Quiet Embryo” hypothesis [30]. Both oocytes and early mouse embryos clearly require a basal level of ATP for normal development [31], very low oocyte ATP content and reduced mitochondrial membrane potential being associated with poor outcome [32]. There appears to be a threshold oocyte mtDNA content that is essential for early development [33] [34] and hence presumably ATP [35]. Our finding that ATP levels are increased in the least successful placenta reflects those of a previous study of early embryos, in which LPD elevated levels of ATP without affecting reactive oxygen species (ROS) production at the two-cell stage [36].

Further, we showed that ATP levels were proportional to fetal water content and inversely proportional to placental efficiency. This outcome is consistent with the highest mtDNA content in the smallest litters that had the lowest overall placental and fetal weights. The poor positive correlation between ATP and mtDNA became significant when compared in small portions of placenta, consistent with wide variation in different regions of the same placenta [24]. It seems likely that mtDNA and ATP content are causally linked.

**Fig 7:**
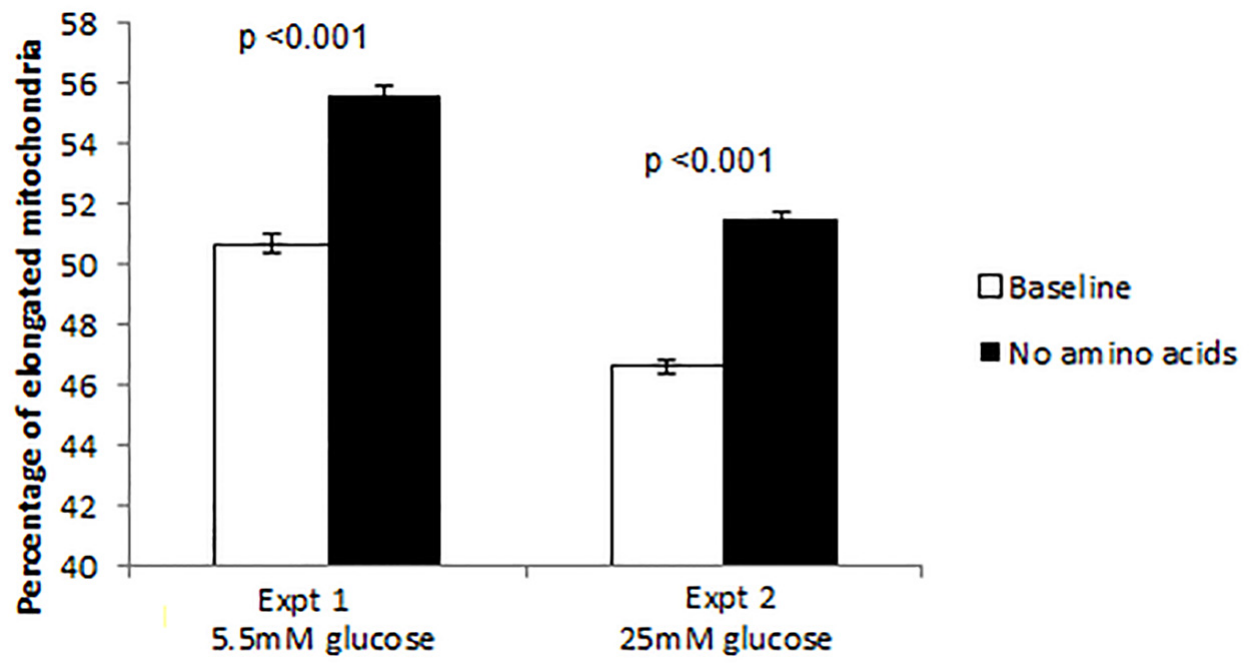
The proportion of elongated mitochondria was significantly greater in cells from the human trophoblast line BeWo, starved of amino acids for up to 24 hours than in control (plot of 2 representative runs out of five, showing the effect of 6 hours amino acid starvation in 5.5mM and 25mM glucose concentrations, both p<0.001 using T tests). Error bars are standard errors.

We suggest three possible explanations for our finding that ATP levels are increased in the least successful placentas. Firstly, low ATP might indicate efficient usage, high ATP perhaps reflecting a mismatch between ATP production and utilisation due to poor delivery. Secondly, active transport may be reduced in LPD placentas, limited not by ATP availability but perhaps by the reduced expression of placental transporters such as *Slc38a2*, *Slc38a4*, potentially regulated by *Igf2* and *Peg3*. Thirdly, a newly characterised response of cultured cells to stress, known as Stress Induced Mitochondrial Hyperfusion (SIMH) [37], that elongates and increases mitochondrial connectivity is able to raise cellular ATP [38]. In tissue culture, amino acid starvation has been shown to cause mitochondrial elongation [25], increased mitochondrial oxygen consumption and increased mitochondrial membrane potential [39] consistent with SIMH. Hence, SIMH could underlie the link between LPD and raised ATP. Normal mitochondrial dynamics could be central to placental development as inner mitochondrial membrane remodelling may play a role in steroidogenesis [40]. We showed that when cultured human trophoblast cells are deprived of amino acids, their mitochondria elongate (Fig 7). Our results suggest that trophoblasts share the SIMH response to amino acid starvation with other cell types, and this may underlie the increase in placental ATP that we observed. Using SIMH to increase cellular ATP may be a particularly appropriate stress response for placenta. The placental syncytium is unlike other cell types, in that it does not need to use mitochondrial fragmentation to drive cell proliferation [41].

SIMH changes mitochondrial organisation and increases cellular ATP by at least two potential mechanisms. Firstly, the highly networked mitochondria seen in SIMH should improve mitochondrial complementation between isolated regions where critical components may be limiting, and hence increase the efficiency of oxidative phosphorylation. In the same way, cells that contain both mutant and normal mtDNA are able to tolerate a high mutant load if mitochondria are networked [42]. Secondly, mitochondrial fission is an important first step in mitophagy, one of several cellular processes that turns over defective mitochondrial components. Hence, mitophagy is inhibited by SIMH [38] and this increases both mitochondrial mass and potentially ATP production. This may be enhanced by increased mitochondrial biogenesis associated with an increased capacity for mitochondrial translation following amino acid starvation [39]. While a mitochondrial stress response may thus confer an epigenetic benefit acutely, it is likely to disadvantage mitochondria by impairing their quality in the longer term. This sequence of events may contribute to the links [43] between increased mtDNA content and ROS of placenta from growth retarded babies [44]. Furthermore, both reduced expression of the pro-mitochondrial protein mitofusin 2 and low ATP levels have been implicated in unexplained early miscarriage [45], where rescue by SIMH may have failed.

Hence, we have preliminary evidence suggesting that increased mitochondrial fusion occurs in trophoblast cells deprived of amino acids. We propose that this reflects SIMH, a protective mechanism that may be occurring in the LPD placenta [45] and in stressed pre-implantation embryos [30].

## Conclusions

LPD was associated with increased and not decreased ATP in placenta, consistent with the quiet embryo hypothesis. We suggest that the mitochondrial stress response, SIMH, may increase the ATP. These findings reinforce the pathophysiological links between maternal diet and *conceptus* mitochondria, operating along different overlapping pathways [46] [47] [48] [49]. Such mechanisms may contribute to the epigenetic regulation of placental phenotype that is important in metabolic programming [29] [50]. Studies using a feeding model that is more relevant to humans would further determine whether the quiet embryo hypothesis extends to placental responses to nutrition [51].

## Supporting Information

S1 Fig Low-protein diet (LPD) impairs weight gain after birth compared with a normal-protein diet. Mouse females subjected to NPD and LPD had their offspring individually weighted (freshly) from ten to 168 days post-natal. Values are reported as means ± SEM. A significant effect of diet (P = 0.0034) and age (P = 0.0001) was observed, but not of the interaction between group and age (P = 0.96). At least six animals per group were considered for each time point.
(TIF)

## References

[1] Roseboom TJ, van der Meulen JH, Ravelli AC, Osmond C, Barker DJ, Bleker OP (2001) Effects of prenatal exposure to the Dutch famine on adult disease in later life: an overview. Mol Cell Endocrinol 185: 93–98. 11738798 10.1016/s0303-7207(01)00721-3

[2] Gluckman PD, Hanson MA, Cooper C, Thornburg KL (2008) Effect of in utero and early-life conditions on adult health and disease. N Engl J Med 359: 61–73. 10.1056/NEJMra0708473 18596274 PMC3923653

[3] Ozanne SE, Martensz ND, Petry CJ, Loizou CL, Hales CN (1998) Maternal low protein diet in rats programmes fatty acid desaturase activities in the offspring. Diabetologia 41: 1337–1342. 9833942 10.1007/s001250051074

[4] Ozaki T, Nishina H, Hanson MA, Poston L (2001) Dietary restriction in pregnant rats causes gender-related hypertension and vascular dysfunction in offspring. J Physiol 530: 141–152. 11136866 10.1111/j.1469-7793.2001.0141m.x PMC2278385

[5] Sibley CP, Turner MA, Cetin I, Ayuk P, Boyd CA, D'Souza SW, et al (2005) Placental phenotypes of intrauterine growth. Pediatr Res 58: 827–832. 16183820 10.1203/01.PDR.0000181381.82856.23

[6] Hanson MA, Gluckman PD (2014) Early developmental conditioning of later health and disease: physiology or pathophysiology? Physiological Reviews 94: 1027–1074. 10.1152/physrev.00029.2013 25287859 PMC4187033

[7] Soejima A, Inoue K, Takai D, Kaneko M, Ishihara H, Oka Y, et al (1996) Mitochondrial DNA is required for regulation of glucose-stimulated insulin secretion in a mouse pancreatic beta cell line, MIN6. J Biol Chem 271: 26194–26199. 8824267 10.1074/jbc.271.42.26194

[8] Wallace DC (2005) A mitochondrial paradigm of metabolic and degenerative diseases, aging, and cancer: a dawn for evolutionary medicine. Annu Rev Genet 39: 359–407. 16285865 10.1146/annurev.genet.39.110304.095751 PMC2821041

[9] Poulton J, O'Rahilly S, Morten KJ, Clark A (1995) Mitochondrial DNA, diabetes and pancreatic pathology in Kearns-Sayre syndrome. Diabetologia 38: 868–871. 7556992 10.1007/s001250050366

[10] Poulton J, Morten KJ, Weber K, Brown GK, Bindoff L (1994) Are duplications of mitochondrial DNA characteristic of Kearns-Sayre syndrome? Hum Mol Genet 3: 947–951. 7951243 10.1093/hmg/3.6.947

[11] von Kleist-Retzow JC, Cormier-Daire V, Viot G, Goldenberg A, Mardach B, Amiel J, et al (2003) Antenatal manifestations of mitochondrial respiratory chain deficiency. J Pediatr 143: 208–212. 12970634 10.1067/S0022-3476(03)00130-6

[12] Owens JA, Falconer J, Robinson JS (1987) Effect of restriction of placental growth on fetal and utero-placental metabolism. J Dev Physiol 9: 225–238. 3611639

[13] Casteels K, Ong K, Phillips D, Bendall H, Pembrey M, The ALSPAC study team, et al (1999) Mitochondrial 16189 variant, thinness at birth, and type-2 diabetes. ALSPAC study team. Avon Longitudinal Study of Pregnancy and Childhood. Lancet 353: 1499–1500. 10232327 10.1016/s0140-6736(98)05817-6

[14] Parker E, Bain S, Cockington R, Phillips D, Poulton J (2004) A common mtDNA variant is associated with high placental weight and thinness at aged 20. Diabet Med 21.

[15] Poulton J, Luan J, Macaulay V, Hennings S, Mitchell J, Wareham NJ (2002) Type 2 diabetes is associated with a common mitochondrial variant: evidence from a population-based case-control study. Hum Mol Genet 11: 1581–1583. 12045211 10.1093/hmg/11.13.1581

[16] Ye Z, Gillson C, Sims M, Khaw KT, Plotka M, Poulton J, et al (2013) The association of the mitochondrial DNA OriB variant (16184–16193 polycytosine tract) with type 2 diabetes in Europid populations. Diabetologia 56: 1907–1913. 10.1007/s00125-013-2945-6 23702607 PMC3737432

[17] Park KS, Chan JC, Chuang LM, Suzuki S, Araki E, Nanjo K, et al (2008) A mitochondrial DNA variant at position 16189 is associated with type 2 diabetes mellitus in Asians. Diabetologia. 10.1007/s00125-008-0933-z 18251004

[18] Armitage JA, Taylor PD, Poston L (2005) Experimental models of developmental programming: consequences of exposure to an energy rich diet during development. J Physiol 565: 3–8. 15695245 10.1113/jphysiol.2004.079756 PMC1464498

[19] Morten K, Field P, Ashley N, Williams KA, Harris D, Hartley M, et al (2005) Fetal and neonatal exposure to AZT and low-protein diet affects glucose homeostasis: a model with implications for AIDS prevention. Am J Physiol Endocrinol Metab 289: E1115–1118. 16014351 10.1152/ajpendo.00226.2005

[20] Moore T, Haig D (1991) Genomic imprinting in mammalian development: a parental tug-of-war. Trends Genet 7: 45–49. 2035190 10.1016/0168-9525(91)90230-N

[21] Suzuki T, Higgins PJ, Crawford DR (2000) Control selection for RNA quantitation. Biotechniques 29: 332–337. 10948434 10.2144/00292rv02

[22] Tosteson DC, Hoffman JF (1960) Regulation of cell volume by active cation transport in high and low potassium sheep red cells. J Gen Physiol 44: 169–194. 13777653 10.1085/jgp.44.1.169 PMC2195084

[23] Heckmann KD, Parsons DS (1959) Changes in the water and electrolyte content of rat-liver slices in vitro. Biochim Biophys Acta 36: 203–213. 14400498 10.1016/0006-3002(59)90085-x

[24] Marchington D, Malik S, Banerjee A, Turner K, Samuels D, Macaulay V, et al (2009) Information for genetic management of mtDNA disease: Sampling pathogenic mtDNA mutants in the human germline and in placenta. J Med Genet. 10.1136/jmg.2009.072900 19914907

[25] Rambold AS, Kostelecky B, Elia N, Lippincott-Schwartz J (2011) Tubular network formation protects mitochondria from autophagosomal degradation during nutrient starvation. Proc Natl Acad Sci U S A 108: 10190–10195. 10.1073/pnas.1107402108 21646527 PMC3121813

[26] Aiken CE, Cindrova-Davies T, Johnson MH (2008) Variations in mouse mitochondrial DNA copy number from fertilization to birth are associated with oxidative stress. Reprod Biomed Online 17: 806–813. 19079965 10.1016/s1472-6483(10)60409-9

[27] Coan PM, Vaughan OR, Sekita Y, Finn SL, Burton GJ, Constancia M, et al (2010) Adaptations in placental phenotype support fetal growth during undernutrition of pregnant mice. J Physiol 588: 527–538. 10.1113/jphysiol.2009.181214 19948659 PMC2825615

[28] Constancia M, Angiolini E, Sandovici I, Smith P, Smith R, Kelsey G, et al (2005) Adaptation of nutrient supply to fetal demand in the mouse involves interaction between the Igf2 gene and placental transporter systems. Proc Natl Acad Sci U S A 102: 19219–19224. 16365304 10.1073/pnas.0504468103 PMC1316882

[29] Fowden AL, Coan PM, Angiolini E, Burton GJ, Constancia M Imprinted genes and the epigenetic regulation of placental phenotype. Prog Biophys Mol Biol. 10.1016/j.pbiomolbio.2010.11.005 21108957

[30] Leese HJ (2002) Quiet please, do not disturb: a hypothesis of embryo metabolism and viability. Bioessays 24: 845–849. 12210521 10.1002/bies.10137

[31] Dumollard R, Duchen M, Carroll J (2007) The role of mitochondrial function in the oocyte and embryo. Curr Top Dev Biol 77: 21–49. 17222699 10.1016/S0070-2153(06)77002-8

[32] Van Blerkom J, Davis PW, Lee J (1995) ATP content of human oocytes and developmental potential and outcome after in-vitro fertilization and embryo transfer. Hum Reprod 10: 415–424. 7769073 10.1093/oxfordjournals.humrep.a135954

[33] Chiaratti MR, Meirelles FV (2010) Mitochondrial DNA copy number, a marker of viability for oocytes. Biol Reprod 83: 1–2. 10.1095/biolreprod.110.084269 20220127 PMC3399397

[34] Larsson N, Wang J, Wilhelmsson H, Oldfors A, Rustin P, Lewandoski M, et al (1998) Mitochondrial Transcription Factor A is necessary for mtDNA maintenance and embryogenesis in mice. Nature Genetics 18: 231–236. 9500544 10.1038/ng0398-231

[35] Johnson M, Freeman E, Gardner D, Hunt P (2007) Oxidative Metabolism of Pyruvate Is Required for Meiotic Maturation of Murine Oocytes In Vivo. Biology of Reproduction 77: 2–8. 17314311 10.1095/biolreprod.106.059899

[36] Mitchell M, Schulz SL, Armstrong DT, Lane M (2009) Metabolic and mitochondrial dysfunction in early mouse embryos following maternal dietary protein intervention. Biol Reprod 80: 622–630. 10.1095/biolreprod.108.072595 19129514 PMC2849812

[37] Tondera D, Grandemange S, Jourdain A, Karbowski M, Mattenberger Y, Herzig S, et al (2009) SLP-2 is required for stress-induced mitochondrial hyperfusion. EMBO J 28: 1589–1600. 10.1038/emboj.2009.89 19360003 PMC2693158

[38] Gomes LC, Di Benedetto G, Scorrano L (2011) During autophagy mitochondria elongate, are spared from degradation and sustain cell viability. Nat Cell Biol 13: 589–598. 10.1038/ncb2220 21478857 PMC3088644

[39] Johnson MA, Vidoni S, Durigon R, Pearce SF, Rorbach J, He J, et al (2014) Amino acid starvation has opposite effects on mitochondrial and cytosolic protein synthesis. PLoS One 9: e93597 10.1371/journal.pone.0093597 24718614 PMC3981720

[40] Wasilewski M, Semenzato M, Rafelski SM, Robbins J, Bakardjiev AI, Scorrano L (2012) Optic atrophy 1-dependent mitochondrial remodeling controls steroidogenesis in trophoblasts. Curr Biol 22: 1228–1234. 10.1016/j.cub.2012.04.054 22658590 PMC3396839

[41] Chen KH, Dasgupta A, Ding J, Indig FE, Ghosh P, Longo DL (2014) Role of mitofusin 2 (Mfn2) in controlling cellular proliferation. FASEB J 28: 382–394. 10.1096/fj.13-230037 24081906 PMC3868832

[42] Malena A, Loro E, Di Re M, Holt IJ, Vergani L (2009) Inhibition of mitochondrial fission favours mutant over wild-type mitochondrial DNA. Hum Mol Genet 18: 3407–3416. 10.1093/hmg/ddp281 19561330

[43] Moreno-Loshuertos R, Acin-Perez R, Fernandez-Silva P, Movilla N, Perez-Martos A, Rodriguez de Cordoba S, et al (2006) Differences in reactive oxygen species production explain the phenotypes associated with common mouse mitochondrial DNA variants. Nat Genet 38: 1261–1268. 17013393 10.1038/ng1897

[44] Lattuada D, Colleoni F, Martinelli A, Garretto A, Magni R, Radaelli T, et al (2008) Higher mitochondrial DNA content in human IUGR placenta. Placenta 29: 1029–1033. 10.1016/j.placenta.2008.09.012 19007984

[45] Pang W, Zhang Y, Zhao N, Darwiche SS, Fu X, Xiang W (2013) Low expression of Mfn2 is associated with mitochondrial damage and apoptosis in the placental villi of early unexplained miscarriage. Placenta 34: 613–618. 10.1016/j.placenta.2013.03.013 23601695

[46] Aiken CE, Ozanne SE (2014) Transgenerational developmental programming. Hum Reprod Update 20: 63–75. 10.1093/humupd/dmt043 24082037

[47] Bruce KD, Cagampang FR, Argenton M, Zhang J, Ethirajan PL, Burdge GC, et al (2009) Maternal high-fat feeding primes steatohepatitis in adult mice offspring, involving mitochondrial dysfunction and altered lipogenesis gene expression. Hepatology 50: 1796–1808. 10.1002/hep.23205 19816994

[48] Igosheva N, Abramov AY, Poston L, Eckert JJ, Fleming TP, Duchen MR, et al (2010) Maternal diet-induced obesity alters mitochondrial activity and redox status in mouse oocytes and zygotes. PLoS One 5: e10074 10.1371/journal.pone.0010074 20404917 PMC2852405

[49] Symonds ME, Pope M, Sharkey D, Budge H (2012) Adipose tissue and fetal programming. Diabetologia 55: 1597–1606. 10.1007/s00125-012-2505-5 22402988

[50] Bressan FF, De Bem TH, Perecin F, Lopes FL, Ambrosio CE, Meirelles FV, et al (2009) Unearthing the roles of imprinted genes in the placenta. Placenta 30: 823–834. 10.1016/j.placenta.2009.07.007 19679348

[51] Symonds ME, Budge H (2009) Nutritional models of the developmental programming of adult health and disease. Proc Nutr Soc 68: 173–178. 10.1017/S0029665109001049 19208271

