# Correction: Is Placental Mitochondrial Function a Regulator that Matches Fetal and Placental Growth to Maternal Nutrient Intake in the Mouse?

**DOI:** 10.1371/journal.pone.0171795

**Published:** 2017-02-03

**Authors:** Marcos R. Chiaratti, Sajida Malik, Alan Diot, Elizabeth Rapa, Lorna Macleod, Karl Morten, Manu Vatish, Richard Boyd, Joanna Poulton

There is information omitted from the “Results” section of the Abstract. The correct “Results” section of the Abstract should read:

Fetal dry weight and placental efficiency (embryo/placental fresh weight) were positively correlated (r = 0.53, P = 0.0001). Individual placental dry weight was reduced by LPD (P = 0.003), as was the expression of amino acid transporter Slc38a2 and of growth factor Igf2. Placental water content, which is regulated by active transport of solutes, was increased by LPD (P = 0.0001). However, placental ATP content was also increased (P = 0.03).

To investigate the possibility of an underlying mitochondrial stress response, we studied cultured human trophoblast cells (BeWos). High throughput imaging showed that amino acid starvation induces changes in mitochondrial morphology that suggest stress-induced mitochondrial hyperfusion. This is a defensive response, believed to increase mitochondrial efficiency, that could underlie the increase in ATP observed in placenta.
