# Supplementary figures and images for: Is Placental Mitochondrial Function a Regulator that Matches Fetal and Placental Growth to Maternal Nutrient Intake in the Mouse?
Source: PLoS One. 2015 Jul 1;10(7):e0130631. doi: 10.1371/journal.pone.0130631 (PMC4488591; doi:10.1371/journal.pone.0130631)

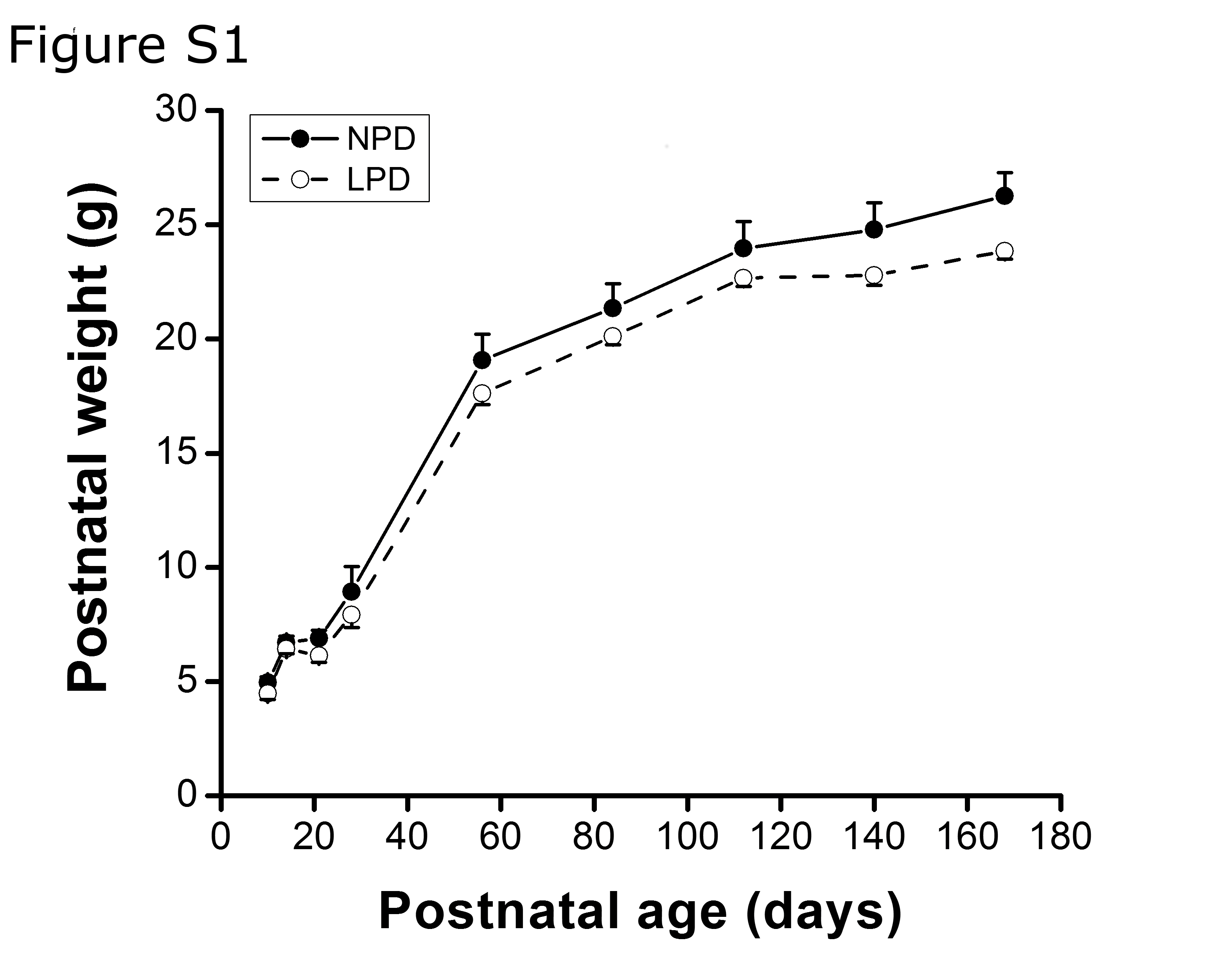

Supplement: S1 Fig — Mouse females subjected to NPD and LPD had their offspring individually weighted (freshly) from ten to 168 days post-natal. Values are reported as means ± SEM. A significant effect of diet (P = 0.0034) and age (P = 0.0001) was observed, but not of the interaction between group and age (P = 0.96). At least six animals per group were considered for each time point. (TIF) [file pone.0130631.s001.tif]
